# A Short Overview of Recent Developments in the Application of Polymeric Materials for the Conservation of Stone Cultural Heritage Elements

**DOI:** 10.3390/ma15186294

**Published:** 2022-09-10

**Authors:** Toma Fistos, Irina Fierascu, Mihaela Doni, Irina Elena Chican, Radu Claudiu Fierascu

**Affiliations:** 1 National Institute for Research & Development in Chemistry and Petrochemistry-ICECHIM, 060021 Bucharest, Romania; 2 Department of Science and Engineering of Oxide Materials and Nanomaterials, University Politehnica of Bucharest, 011061 Bucharest, Romania; 3 Faculty of Horticulture, University of Agronomic Sciences and Veterinary Medicine of Bucharest, 011464 Bucharest, Romania

**Keywords:** stone heritage, polymeric materials, weathering, hydrophobic materials, superhydrophobic materials, superamphiphobic materials

## Abstract

Stones are ones of the most ancient natural materials exploited by humans, with different uses, from tools to buildings, that have endured over time in better conditions than other objects belonging to cultural heritage. Given the importance of those silent witnesses of our past, as well as our duty to preserve all parts of cultural heritage for future generations, much effort was put into the development of materials for their consolidation, protection, self-cleaning, or restoration. Protection of ancient stone monuments and objects has gained the interest of researchers in the last decades in the field of conservation of cultural heritage. In this respect, the present paper aims to be a critical discussion regarding potential polymeric materials, which can be used in restorative and conservative approaches for stone materials of cultural heritage importance, against physical degradation phenomena. Recent advances in this area are presented, as well as the current bottle-necks and future development perspectives.

## 1. Introduction

Stones are one of the most ancient natural materials exploited by humans, with different uses, from tools to buildings, that have endured over time in better conditions than other objects belonging to cultural heritage. Even though they are materials with a good durability, some external factors can deteriorate it, and keeping ancient proof of civilization for future generations can pass in a “mist of time”. Natural stones are not only a material resource, they are also cultural. They allow us to understand different populations’ way of life, beliefs, and values [1]. 

Over time, different types of stone were used to manufacture small objects, which served as tools, vessels, jewelries, or weapons [2,3,4] or for big construction projects, such as roads or buildings, defining the architectural identity of the zone [5,6,7]. The characteristics of the materials used thousands of years ago reveal to us in the present the dynamic of the populations, transport methods, and way of living of our ancestors [8]. In addition, using modern characterization methods, some approaches for conservation and restoration can be considered, due to their intrinsic features. Their mineralogical properties or microstructural characteristics can affect physical and mechanical behaviors [9]. The relationship with microbial colonization and development of biodeterioration and damage is also related to stone characteristics [10]. 

Surviving stone monuments for the future is our duty, so by addressing different conservation and restoration methods, we can slow down the deterioration process. If, in the past, stone has always been considered the most affordable and durable material, nowadays ancient stone objects belonging to cultural heritage, due to the carried cultural load, are in need for modern conservation and protection materials and technologies. 

In this respect, the present paper aims to be a critical discussion regarding potential polymeric materials, which can be used in restorative and conservative approaches for stone materials of cultural heritage importance, against physical degradation phenomena. 

The recent advances in this area are presented, as well as current bottle-necks and future development perspectives.

## 2. Deterioration of Natural Stones

When speaking of stone heritage, two main classes can be distinguished: natural stones (that can be further classified into inclusive rocks—i.e., granite, diorite, gabbro, etc., extrusive rocks—i.e., basalt, andesite, rhyolite, etc., sedimentary rocks—i.e., sandstone, limestone, gypsum, etc., and metamorphic rocks–marble) and man-made materials (such as fired or unfired bricks). A thorough classification of the stone materials (including their composition, characteristics, and uses) was previously presented by our group [11]. 

**Figure 1:**
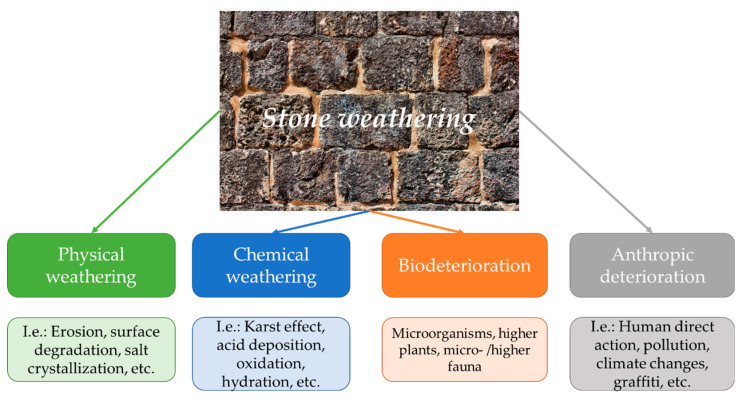
Types of stone weathering.

Although having the appearance of durable materials (and often resisting for hundreds or thousands of years), the cultural heritage stones are exposed to degradation, either from natural or anthropic factors. In the following paragraphs we will briefly discuss some of the factors involved in the weathering of stone materials, underlining the necessity for developing tailored materials for their conservation. It must be stated that all types of weathering are in a strong connection, acting in a synergistic manner (Figure 1).

### 2.1. Physical Weathering

The phenomenon of physical weathering is common for most types of stone materials, being caused by natural agents (especially water, but also wind or temperature variations) [12]. One of the major types of physical weathering is the superficial erosion, determined by a combination of factors, such as rainfall, winds, and presence of particles that can act as abrasive agents. The presence of water can also lead to surface degradation of stones through expansion/contraction or freeze/thaw cycles, generating cracks, scaling, exfoliation, spalling, delamination, or contour scaling [13,14,15,16]. 

Most commonly, these processes are associated with porous stones (especially sedimentary rocks, such as limestone or sandstone, and man-made materials) [17] and to a lesser extent with the stones having superior mechanical properties (i.e., granites) [12]. 

Salt crystallization represents another common process responsible for physical weathering. Salt solutions (originating from the structure of the stone, plasters, soil–ions migrating using the capillarity of the stones, anthropogenic activities, such as agricultural practices, deicing solutions or even materials used for conservation of the artifacts, atmospheric pollution, metabolic products of microorganisms, etc.) can increase in concentration and finally crystalize with the reduction of relative humidity. With the increase in relative humidity, the crystals are rehydrated, and thus repeated crystallization/re-hydration cycles occur, which can lead to an increased mechanical stress on the stone, thus causing its weathering [18]. 

Recent works [16] revealed that most of the physical weathering phenomena recorded are in a strong connection with the presence of swelling clays, zeolites or micropores in the composition of the stones.

Another type of physical weathering is represented by the action of plant roots, which can be developed in the existent cracks and exert further damage [19].

### 2.2. Chemical Weathering

The chemical weathering of stone artefacts represents the alteration of the stone composition caused by chemical reactions. One of the most common chemical weathering, particularly affecting the calcareous stones is represented by the karst effect [12,20]. The karst effect represents, basically, the chemical transformation of calcium carbonate to the highly soluble calcium bicarbonate. The effect is aggravated by the presence of pollution related CO_2_ and can lead to an increase of stone’s pH, by the formation of carbonic acid and subsequent processes, which are necessary for the re-establishment of the equilibrium [12]. 

The acid deposition, either wet (trough acid rain) or dry (through the deposition of pollutants, such as SO_2_ or NO_2_) leads to the formation of acidic species and their reaction with the stone’s components, subsequently forming soluble compounds, which are easily removed from the surface of the stone. As a new layer is exposed, the process is once again initiated and the stone is irremediably damaged. The process is often present in calcareous stones in which it leads to the formation of gypsum (CaSO₄ × 2H₂O). Although not as sensitive as the porous stones, the highly crystalline ones (such as granite or marble) are also affected by this process. In these cases, it leads to the apparition of efflorescence, but also of a porous layer, which enables the apparition of otherwise specific to porous stones physical degradation phenomena [13,20].

The oxidation phenomenon (mostly encountered as the oxidation of Fe^2+^ to Fe^3+^) can affect a very wide range of materials, practically any type of stone, with a minor content in any oxidation-prone metal, being exposed to the formation of oxidation stains (brittle, affecting the mechanical properties of the stone) in the presence of oxygen and water, including granite [21], marble [22], or limestone [23]. 

The hydration of particular minerals present in the stone structure does not represent in itself a major treat to the object’s integrity, but it represents an initial step in the hydrolysis process [16]. Common examples of the hydration process are the hydration of the iron oxides to hydroxides or of anhydrite to gypsum [24]. 

### 2.3. Biodeterioration

Although, from a microbiological point of view, stones represent a very poor growth media, the biodeterioration, or degradation of stone materials induced by (micro)organisms, is encountered on all types of substrates and in all climates, the exact type of colonizing species being influenced by the bioreceptivity of the stone (predisposition of a particular material to be colonized by a living organism) [25]. Bioreceptivity is a particular characteristic of each cultural heritage site, as it is dependent on the stone structure, petrophysical characteristics, chemical composition, pH, conservation state, weather conditions, or air pollution [25]. 

Regarding the colonizing organisms, there are several classifications currently used: following a nutritional classification, the organisms inducing biodeterioration can be divided into photoautotrophs, chemoautotrophs, heterotrophs and chemoorganotrophs [25,26]. From a taxonomic perspective, the biodeteriogens can be divided into bacteria, archaea, cyanobacteria, algae, fungi, and lichens [11,26]. The biodeteriogens can also be divided into microorganisms, higher plants, and micro- and higher fauna [11]. 

The microorganisms-induced biodeterioration is manifested in a very wide variety of effects, including dissolution/recrystallization, biofilm development, chemical alterations, discolorations, etc., while the deterioration induced by higher organisms is usually associated with physical effects, such as erosion or apparition/deepening of cavities [11]. The biodeterioration represents by itself a subject of intense research, the causes, specific species affecting different types of stones, effect and treatment methods being discussed in a large number of review papers [25,26,27,28,29,30,31].

### 2.4. Deterioration Induced by other Anthropic Factors

As previously presented, the anthropic factors can be involved in all the deteriorations processes. Other human actions can also contribute to the degradation of stone artefacts. For example, the moisture originating from ineffective systematization, clogged drains or installation system failure can affect the stone materials to a great extent [32]. The contribution of human activities to the pollution and involvement of the pollutants in the chemical and physical degradation processes represents another important degradation factor [12], as are the activities involving the enrichment of soil found in contact with the stone artefacts in nitrates or chlorides [18], or incorrect/unsupervised conservation and restoration treatments attempts. 

Climate changes can also affect the stones, not only by accelerating certain degradation processes, but also by rendering ineffective previously applied conservation materials [33]. Extreme events (such as fire) can induce a rapid and acute decay of the stone, triggering differential thermal expansion of different materials, fracturing, spalling or materials loss, as well as long-term effects, such as micro-cracks or changes in surface composition leading to further decays [34]. 

Another deterioration factor specific to the Anthropocene is represented by the graffiti. Present all around us, graffiti affect the stone of cultural importance and are particular difficult to counteract, as the graffiti associated materials include a series of agents (paints, polyurethanes, lacquers, enamels, chalk, lipstick, wax, adhesives, etc.) that induce most often a chemical degradation but also the physical decay of the stone [35]. More than that, the removal of the graffiti can lead to subsequent degradations, including the use of abrasive materials that leads to surface alterations, chemical contamination, or mineralogical alterations [36].

## 3. Recent Advances in Polymeric Materials for the Protection of Stones of Cultural Importance

As previously presented, the stone materials are affected by a multitude of factors, leading to their decay. Given the importance of those silent witnesses of our past, as well as our duty to preserve as much parts of the cultural heritage as possible for future generations, much effort was put into the development of materials for their consolidation, protection, self-cleaning or restoration. As the inorganic materials and nanomaterials are a major subject in this area, multiple advances being recorded, they are also subject of multiple very competent and periodic review works emphasizing their importance and potential advantages in application [37,38,39,40]. 

**Figure 2:**
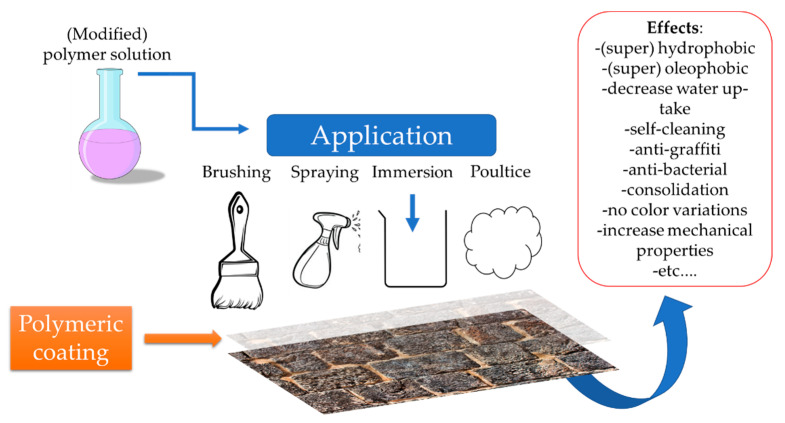
Polymeric coatings for the protection of cultural heritage stones (overview of reviewed results).

As presented in the previous section, water presence represents a determining factor for all the types of stone weathering. As such, the development of water-repellent or moisture control treatments is one of the main desiderates of conservation science. In this area, polymer science could contribute to the development of tailored materials, designed according the stone characteristics. Considering the lack of up-to-date information, the goal of the present review, to be elaborated in the following paragraphs, is to present the recent progresses in the development and application of polymeric materials in this specific area (Figure 2).

### 3.1. Hydrophobic Coating Materials

Coating material are a simple solution to preserve and protect surfaces from weathering phenomena. A special requirement for these materials is to not affect the surface of the heritage object over time. The emerging demand to reduce the emission of volatile organic compounds (VOC) led the interest of the researchers to develop new types of coatings. In Table 1 are presented some results from specific literature with emphasis on “environmentally friendly” materials.

Sbardella and coworkers demonstrated that polyacrylate/silica hybrid is a good candidate as a coating material for stone artefacts. The addition of nano-silica in the polymer created good hydrophilic/hydrophobic balance and enhanced the mechanical scrub resistance [41]. Furthermore, after accelerated photoaging was demonstrated that silica nanoparticles affected the capillary absorption behavior compared to the untreated stones thus reducing water absorption. The values obtained for the treatments demonstrated that these materials have generally hydrophobic behavior, reducing the absorption of water by capillary effect. 

Reversibility is also a mandatory requirement for materials used in heritage conservation. Coatings based on poly(hydroxyalkanoate)s were used by Andreotti and coworkers as coatings for sandstone (Siena stone), limestone (Lecce stone), and marble (Carrara marble) [42]. They demonstrated the performance and compatibility of the protective hydrophobic treatments in terms of capillary water absorption, static and dynamic contact angles, surface tension, water vapor diffusion, color alteration, and surface morphology. The ability to reduce the capillary water absorption represents a significant parameter, which could be used for predicting the on-site performance of the treatment. In the case of this study, limestone presents a relatively high water absorption capacity (final water uptake ~305 kg/m^3^), compared to sandstone (final water uptake ~93 kg/m^3^, probably due to significantly higher open porosity and the coating treatment presents good results (mean ratio of protection after 48 h reaching 96% for polymer applied by dip coating). The same parameter reached 92% for sandstone after poultice treatment with polymer. The results obtained were compared with commercial, widely applied treatments (a silane and siloxane solution, respectively a mixture of silane and siloxane emulsified in water), the proposed materials for treatment having superior results for some of the tested parameters. 

Colangiuli et al. [43] evaluated the efficiency of TiO_2_ NPs/fluoropolymer coatings applied on limestone buildings kept in urban environment. The authors performed contact angle measurements, capillary water absorption tests, and self-cleaning efficiency evaluation (using photodegradation test of Rhodamine B) [43]. In this case, self-cleaning efficiency was found to depend on the titania contents used in the mixtures (the experimental variants studied containing 11, respectively 50% NPs), but the mixture itself could present in time an increased damage risk for the stone due to the coupling of the photocatalytic titania with the hydrophobic polymer that led to low contents of water-soluble ions adsorbed by the NPs, which may be accumulated on the coated stone surface. 

Such polymeric coatings are also valuable to repel both water and oil and act as an anti-graffiti barrier, as demonstrated by Lettieri et al. [44], with superior results (in terms of contact angles) compared with other commercial products, but, the polymer itself cannot be used, without adding nanoparticles, as the pure acrylic coating undergoes severe yellowing [45]. The global color differences recorded for the treated samples after ageing tests were as low as 1.4 (compared with the untreated sample 1.7), but the color difference recorded for SiO_2_ sample was 9.7 [45]. 

The protective effects against bird and bats droppings, also known as guano, was studied by Lettieri and coworkers for a polymer coating containing SiO_2_ nanoparticles in comparison with two commercial protective polymer products (commercial fluoropolyether, and a commercial silicon-based polymer) [46]. After treatment and pancreatin test, the increased values of contact angle (144° after treatment, compared with 35° before treatment) suggested the presence of pancreatin residues in the pores. In the same time, the protected samples exhibited smaller color variations in comparison to the unprotected control surfaces but also by comparison with the commercial products. Regardless the coating used, the original color of the stone was not regained in the cited study. Besides contact angle test, “contact sponge test” can prove the efficiency of the coating in order to control the surface hydrophobicity [51]. In some cases, hydrophobic properties of the coating present antimicrobial properties too [47]. Sometimes, added photocatalytic nanomaterials might generate excess free radicals to degrade the polymer matrix conducting to a reduction of water adsorption capacity which is not suitable for stone heritage relics’ protective coating [52]. In addition, the yellowing process of the polymer is given its chemical structure, where radicals are able to start photochemical reactions [53]. Corcione and coworkers, using a treatment based on acrylic resin, silanes, and nano-particles of boehmite, recorded good results for calcareous and porous stones, such as Pietra Gentile and Pietra Leccese, the protective material being able to harden at room temperature in short times if exposed to UV-radiations [49].

Another approach for the development of hydrophobic materials is represented by the modification of commercial products. Harvesting the advantages of an established product (such as proven efficiency, or the large-scale acceptance of its application) with the benefits of newly developed materials, this approach can shorten the path from laboratory research to practical application. Li et al. [54] explored the possible application of a methyl-modified silica hybrid fluorinated Paraloid B-72 coating. The modification of the commercial product with 20% fluorinated polymers and 12% nano-silica grafted by hexamethyldisilazane led to the development of a hydrophobic coating on ancient bricks (contact angle 142.3°) with a reduced porosity (34.68%, compared with 38.88% for the untreated sample and 36.89% for the fluorinated polymer), and increased acid, salt and alkali resistance. Additionally, the presence of the SiO_2_ nanoparticles increase the UV-resistance of the polymers, although their degradation could not be avoided.

A 3-year in situ evaluation of the consolidation properties of a polymer-based treatment (monomeric and oligomeric ethoxysilanes with SiO_2_ nanoparticles) performed on bioclastic sandstone demonstrated the good performance of the consolidant (increased mechanical resistance, uniform penetration, with minimum effects on the vapor permeability and chromatic variation [50].

### 3.2. Superhydrophobic and Superamphiphobic Coating Materials 

In the last two decades, the interest of the researchers to develop new material with hydrophobic properties, especially superhydrophobic (contact angle of water drop, WCA > 150°) and superamphiphobic (WCA > 150° and contact angle of oil drop, OCA > 150°) gained a great interest [55]. Cassie and Baxter (1944) stated almost eighty years ago that “*The duck is generally regarded as having attained perfection in water repellency, and it is usually taken for granted that the duck uses an oil or similar coating with larger contact angles than any known to man. In actual fact, the duck obtains its water repellency from the structure of its feathers*” [56] thus conducting to a relation from the structure of the support and wetting phenomena.

The new trends in development of material for conservation and restoration cultural heritage stone objects are given by these superhydrophobic materials, which are in principle, advantageous over the typical hydrophobic coatings [57]. Super-oleophobic and oil repellent materials are suitable to protect stone artefacts of the cultural heritage, which are threatened by oil-based pollutants, particularly in urban areas [58]. Cappelletti and coworkers applied a commercially available Si-based resin (Alpha^®^SI30, a polysiloxane) on Carrara/Botticino marbles and Angera stone, in order to improve the hydrophobicity features of the surfaces themselves, obtaining θ > 150° (for Carrara) and for Angera and Botticino samples (138° < θ < 141°) by adding TiO_2_ [59]. In this respect, the obtain hybrid coatings were more effective in reducing salts formation rather to the pure resin. Furthermore, by adding TiO_2_, ZnO, and Ag into silane monomers applied on Ajarte limestone, Gherardi and collaborators obtained good results through a distribution inside the substrate and a suitable coverage of the pore walls [60]. Tian et al. obtained superhydrophobicity (WCA > 150°) at increased concentrations of TiO_2_ coated SiO_2_ added in fluorinated siloxane polymer and applied on sandstone [61]. Other nanoparticles (such as Al_2_O_3_, ZnO or SnO_2_) can be used in different polymeric matrixes in order to obtain superhydrophobic properties [62,63,64]. Pure polymers can be used as superhydrophobic materials, only when the polymer has a low surface energy and the stone surface has high roughness, and WCA > 150° can be achieved [65,66,67].

By a simple definition, the superamphiphobic materials have the ability to repel not only water but also liquids with lower surfaces tensions, including oil. It is challenging to obtain them, as it is more difficult to impede the wetting of low surface tension liquids, such as oil, than water [68]. By adding different concentration of SiO_2_ nanoparticles in the Silres BS29A emulsion (commercially available, composed of silane, siloxane, and organic polymer), OCA started to decrease, suggesting that the oil drops sank into the grooves [69]. By enriching fluorine resin with SiO_2_, Lettieri and coworkers achieved OCA ∼ 122° on calcareous stones after the treatment [70]. Mosquera’s group obtained good results after a treatment of granite with fluoroalkylsilanes-SiO_2_ composite: contact angle > 150°, minor color differences after self-cleaning tests (ΔE 0.37–0.51), high resistance to rain water [71].

### 3.3. Polymer Incorporation in Other Materials 

Another important aspect regarding the preservation of cultural heritage stones, especially when speaking of buildings of cultural importance is represented by the mortars, cements and grouts. Development of materials compatible with the stones is essential for a successful restoration [72].

Different types of synthetic polymers (acrylates, acetates, polyvinyl alcohol, etc.) can be incorporated in mortars, leading to the increase of mechanical properties, as well as ion migration properties, acid attack resistance, or freeze–thaw resistance [73,74,75,76]. On this topic, the presence of a series of patents (some decades old) represents an argument for a ready-to-market solution, which could represent an important instrument for restorers [77,78,79,80]. 

Impregnation of limestone with different grades polyethylene glycol (PEG) led to the development of phase change materials, which, incorporated in mortars, resulted in materials with appropriate workability, flexural, and compressive strength [81,82,83]. The advantage of the proposed solutions resided in the possibilities to use the mortar both in cold and warm climates, due to large intervals of melting/crystallization temperatures. 

Nývlt et al. [84] studied the characteristics of various waterproofing screed for protecting different type of materials (ceramics, concrete, lime-sand bricks, marl stones). From all the tested screeds (which were all confirmed as viable waterproofing materials), the polymer-based one (containing polyurethanes, epoxy and polyester) showed the best cohesion to all the substrates, although exhibiting the lowest durability (the cohesion being greatly affected by the freeze-thaw cycles). 

## 4. Concluding Remarks and Possible Developments

The field of cultural heritage represents not only an important socio-economic resource [85], but its current state of preservation also represents an indicator of the civilization and awareness of a particular nation [86]. Unfortunately, regardless of their particular composition, the artifacts are subject to degradation processes. Among the most important elements affecting the cultural heritage and its transfer to future generations can be considered the environmental factors (light, temperature, relative humidity), anthropogenic causes (pollution, inappropriate restoration interventions, vandalism), biocontamination, natural disasters (floods, fires), and climate changes [87]. In the last decade, the specialists in this field proposed a series of innovative materials and methodologies, applied in different areas, including the diagnosis and conservation state monitoring [88,89] or preventive measures, implemented in order to reduce the need for interventions [90,91]. Thus, thorough scientific studies are needed to develop a tailored formulation in order to protect, preserve, and restore cultural heritage objects, and this represents a continuous challenge for the scientists, aiming to replace the current serendipitous approaches in restoration [87]. 

The main bottlenecks in developing new materials for the protection of cultural heritage are related to the properties of the objects after the treatment. Any treatment applied to cultural heritage artifacts should meet several critical conditions, among which two are of particular interest: the reversible character of the treatment and authenticity of the preservation [92]. Some limitations of current approaches related to the properties of the treatments thus appear: effectiveness, durability, penetration, absence of visible interfaces between the treated and untreated areas; maintenance of stone porosity to allow its perspiration and water circulation; chemical compatibility, avoiding chemical reactions or the formation of layers on the substrate or altering the aesthetic aspect, both in its color and its brightness. Besides, the treatment must maintain its properties over time, without deteriorating due to the effect of external agents.

The commercial polymeric materials, extensively used for the protection of cultural heritage stones since the 1960s, raises several problems, as they are unable to meet the previously stated conditions: they usually suffer irreversible alteration over time (affecting their conservative properties), and suffer color changes overtime, while their complete removal is often impossible. In addition, the decreased porosity and permeability of stone causes the nucleation and growth of salts at the interface between the polymer layer and the stone surface, leading to the flaking and disaggregation of the stone surface layers and the disruption of the polymer coating [93,94]. On the other hand, the current concerns for environmental protection requires the development of new materials, having an environmentally friendly character [51].

The question arising from these problems is *what can be done?* In our opinion, the polymeric materials should not be abandoned. Developing new organic/inorganic composites, based on environmentally friendly materials, could overcome some of the shortcomings of the commercial materials [95], while also preserving the short-term performances of the polymeric conservation materials designed for stone artefacts. This approach could also overcome the relative and understandable reluctance of conservators towards the introduction in practice of new materials.

The solutions recently developed (and reviewed in the present work) are able to harvest the advantages of the polymers, and enhance them with self-cleaning, antimicrobial, consolidant capacities induced by other types of materials. The reviewed materials preserve their characteristics over longer periods of time, suffers lesser color changes, and affects in a lesser extent the porosity and permeability of stone, while enhancing their mechanical properties, using different types of deposition techniques (brushing, spraying, dip coating) [55,96]. 

Further research is necessary in this area, for the long-term evaluation of their effects on the stone substrate, as well as for the continuous development and evaluation of new treatments for cultural heritage stone preservation. 

## Figures and Tables

**Table 1:** Recent developments in the hydrophobic coating materials ^1^.

Coating Material	Support Material	Stone Characteristics	Application	Solvent/Application Method	Results	Ref.
Polyacrylate/silica hybrid (SiO_2_ nanoparticles)	Lecce stone, Carrara marble	Not provided by the authors	Decreasing water penetration (water up-take by capillary adsorption)	Waterborne miniemulsion, applied by brushing	AC = 10.90–13.65 kg/m^2^ × h^0.5^ (Lecce stone, control 108.5), 0.07-0.10 (Carrara marble, control 54.08); RCI = 0.78–0.85 (Lecce stone); 0.60–0.94 (Carrara marble), control = 1; θ = 88.93–93.95° (Lecce stone, control = 0), 88.28-94.60° (Carrara marble, control = 54.08)	[41]
Poly(hydroxyalkanoate)s(PHBVV and PHB)	Sandstone (Siena stone), limestone (Lecce stone) and marble (Carrara marble)	P: 0.1–10 µm; calcite: 88% in sandstone, 86% in limestone and 98% in marble; 2% dolomite (in marble), quartz (in sandstone), fluoropatite (in limestone), by XRD analysis	Decreasing water penetration (water up-take by capillary adsorption)	Solvent-chloroform, applied by dip coating, poultice, spraying	Best results: sandstone—R_p_ after 48 h—86–92% (all PHBVV treatments, commercial treatment 89–97%, dip coating and spray); limestone—R_p_ after 48 h—91–96% (all PHBVV treatments, commercial treatment 87–95%, dip coating and spray); θ = 123 ± 0°—poultice PHBVV (control 15 ± 4°); Limestone θ = 126 ± 7°—poultice PHBVV (control 0 ± 0°); marble θ = 109 ± 10°—poultice PHBVV (control 41 ± 7°); WVT (g/m^2^ day) = 59—PHB spray, 60—PHBVV dip coating (sandstone, control 86); 126—PHBVV dip coating (limestone, control 278); 11—PHB spray (marble, control 21)	[42]
TiO_2_ NPs/fluoropolymer, at 11, respectively 50% NPs	Limestone	Not provided by the authors	Hydrophobic and self-cleaning coating	Water dispersion, applied by brushing	D = 90%/95%; ΔE after 1 year = 1.01/2.46 (2.96, without NPs); Contact angle: >100° before exposure, 50–80° after 1 year of exposure and washing	[43]
Fluorine resin containing SiO_2_ NPs	Calcareous stones (porous calcarenite, compact limestone)	Calcarenite: calcite (93–97%), P = 39%, pore size: 0.5–6 μm; limestone: calcite (>95%), clay, iron oxides, P = 2%, pore size: 0.025–0.001 μm	Anti-graffiti barrier	Water dispersion, applied by brushing	Calcarenite: WCA 139°, OCA 114° (control 40/13, commercial products: 106–114/56–93); Limestone: WCA 142°, OCA 122° (control not determinable, commercial products: 119–122/56–114); ΔE after staining and cleaning—comparable with the commercial products	[44]
(3-(trimethoxysilyl)propyl methacrylate containing 2–10% silica	Carrara Marbleand Lecce stone	θ = 30–79°; Young’s modulus(MPa) = 8–122	Reducing water absorption,	Waterborne coating, applied by brushing	θ = up to 94°, dependent on silica modification matrix and silica content; Young’s modulus (MPa—nt), Tensile Strength (MPa)—up to 9.60, water uptake—10–70% ΔE = 1.4 for the methanol modified silica, 5% silica coating (untreated sample = 1.7)	[45]
Fluorine resin containing SiO_2_ NPs	Calcareous stones (porous calcarenite, compact limestone)	P = 42/1.98, pore radius 1.23/0.010 μm, pore size 0.5–4/0.01–0.03 μm. Initial colorimetric parameters (L*, a* and b*): 80.33; 1.42; 16.45/83.87; 1.20; 6.03	Guano protective layers	Waterborne coating, applied by brushing	G [(g/h)∙10^−3^] = 18.7/4.0 WVT (g/m^2^∙day) = 230/58; ΔVP after pancreatin test = −22/−30 (control −34/−68); ΔE = 0.96/1.65 (control 3.97/4.29, commercial products 2.10, 3.92/1.70, 3.43), WCA = 144/141°	[46]
Sodium polyacrylate (NaPAC16); MgO, and respectively TiO_2_ composite	Mosaic stone (limestone and marble)	Periclase and anatase (XRD), total pore volume (cm^2^/g)—0.16–0.68, pore diameter (nm)—6.04–33.1	Antibacterial and hydrophobic coating	Water dispersion, applied by immersion	Reduction of OD (*Staphylococcus aureus)*, IZ = 11/14 (*S. aureus),* 9/6 (*Aspergillus niger*), 7/4 (*Candida albicans);* θ = 106/107, ΔE < 1 for all samples and stones treated	[47]
ZrO_2_-doped-ZnO-PDMS	Lecce stone, brick, and marble	Not provided by the authors	Protection and self-cleaning effect	Solvent-ethanol, applied by brushing	Qf = 479.04 ± 8.16 mg cm^−2^ (Lecce stone), Qf = 346.66 ± 10.49 mg cm^−2^ (brick), and Qf = 15.34 ± 1.60 mg cm^−2^ (marble) D* = 6.05–72.25%	[48]
Acrylic resin (TMPTMA), silanes (MEMO) and nano-particles of boehmite	Calcarenitic stones (Leccese stone and Gentile stone)	P = 33.5/21.9, bioclasts size 150/200 μm	Water repellent	Trimethylolpropane trimethacrylate base, applied by brushing	θ = 130/118°, ΔE = 6.8/3.6, PE = 68/52%	[49]
Monomeric and oligomeric ethoxysilanes with SiO_2_	Ostionera stone (bioclastic sandstone)	WAOP = 27.8%, WCs = 13.5% WPP = 4.5 × 10^−6^ m^2^/s	Consolidation, in situ application	Water dispersion, applied by brushing	Increase of mechanical properties (>25%), WPP—6% decrease, LWM-19% decrease, ΔE = 1.5, PD	[50]

^1^ Where: AC—absorption coefficient, RCI—relative capillary index, θ—contact angle, PHBVV—poly(3-hydroxybutyrate-co-3-hydroxyvalerate-co-4-hydroxyvalerate, PHB—Poly(3-hydroxybutyrate, P—porosity, XRD—X-ray diffraction, R_p_—mean ratio of protection, WVT—water vapor transmission rate, NPs—nanoparticles, D—photodegradation activity, ΔE—global color difference, L*—lightness parameter in the CIELAB color space, a*—chromaticity coordinate for the red-green component in the CIELAB color space, b*—chromaticity coordinate for the blue-yellow component in the CIELAB color space, WCA—water-stone static contact angle, OCA—oil-stone static contact angle, G—water vapor flow rate, ΔVP—vapor permeability variations, OD—optical density, IZ—inhibition zone, PDMS—polydimethylsiloxane, Qf—absorbed amount of water by the capillary method, D*—discoloration factor; TMPTMA—trimethylolpropane trimethacrylate, MEMO—trimethoxypropylsilane methacrylate monomer, PE—protective efficacy, WAOP—water accessible open porosity, WCa—water content under atmospheric pressure, WPP—water vapor permeability, LWM—liquid water movement by capillarity, PD—penetration depth—30 mm.

## Data Availability

Not applicable.
